# Biphasic calcium phosphate doped with zirconia nanoparticles for reconstruction of induced mandibular defects in dogs: cone-beam computed tomographic and histopathologic evaluation

**DOI:** 10.1007/s10856-023-06731-5

**Published:** 2023-05-19

**Authors:** Said K. Taha, Elham A. Hassan, Sahar Mousa, Gehan T. El-Bassyouni, Heba N. Shalash, Mohamed A. Abdel Hamid

**Affiliations:** 1 Surgery and Oral Medicine Department, Oral and Dental Research Institute, National Research Centre, 33 El Buhouth St., Dokki, Giza, 12622 Egypt; 2 Department of Surgery, Anesthesiology and Radiology, Faculty of Veterinary Medicine, Cairo University, Giza, 12211 Egypt; 3 Inorganic Chemistry Department, Advanced Materials Technology and Mineral Resources Research Institute, National Research Centre, 33 El Buhouth St., Dokki, Giza, 12622 Egypt; 4 Refractories, Ceramics and Building Materials Department, Advanced Materials Technology and Mineral Resources Research Institute, National Research Centre, 33 El Buhouth St., Dokki, Giza, 12622 Egypt; 5 Basic Dental Science Department, Oral and Dental Research Institute, National Research Centre, 33 El Buhouth St., Dokki, Giza, 12622 Egypt

## Abstract

The present study aimed to evaluate osteogenic potential and biocompatibility of combining biphasic calcium phosphate with zirconia nanoparticles (4Zr TCP/HA) compared to biphasic calcium phosphate (TCP/HA) for reconstruction of induced mandibular defects in dog model. TCP/HA and 4Zr TCP/HA scaffolds were prepared. Morphological, physicochemical, antibacterial, cytocompatibility characterization were tested. In vivo application was performed in 12 dogs where three critical-sized mandibular defects were created in each dog. Bone defects were randomly allocated into: control, TCP/HA, and 4Zr TCP/HA groups. Bone density and bone area percentage were evaluated at 12 weeks using cone-beam computed tomographic, histopathologic, histomorphometric examination. Bone area density was statistically increased (*p* < 0.001) in TCP/HA and 4Zr TCP/HA groups compared to control group both in sagittal and coronal views. Comparing TCP/HA and 4Zr TCP/HA groups, the increase in bone area density was statistically significant in coronal view (*p* = 0.002) and sagittal view (*p* = 0.05). Histopathologic sections of TCP/HA group demonstrated incomplete filling of the defect with osteoid tissue. Doping with zirconia (4Zr TCP/HA group), resulted in statistically significant increase (*p* < 0.001) in bone formation (as indicated by bone area percentage) and maturation (as confirmed by Masson trichrome staining) compared to TCP/HA group. The newly formed bone was mature and organized with more trabecular thickness and less trabecular space in between. Physicochemical, morphological and bactericidal properties of combining zirconia and TCP/HA were improved. Combining zirconia and TCP/HA resulted in synergistic action with effective osteoinduction, osteoconduction and osteointegration suggesting its suitability to restore damaged bone in clinical practice.

**Graphical Abstract:**

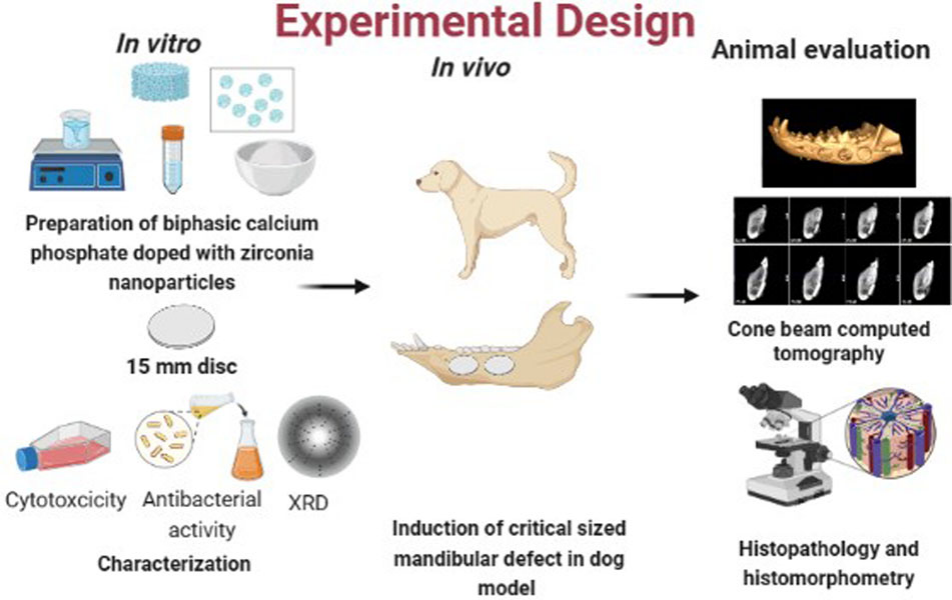

## Introduction

Bone defects resulting from trauma, tumor resection, surgical complication, or congenital diseases represent major challenge for orthopedic and maxillofacial surgeons [1, 2]. Bone grafting procedure remains the gold standard for replacing the damaged bone. However, donor site shortage and morbidity, the need for additional surgery, prolonged healing time and the risk of disease transmission are limiting factors [1].

Matrix-based bone tissue engineering strategies aim to replace the damaged bone with structural implants/material (scaffolds) to recruit the endogenous osteoinductive factors and attract endogenous osteogenic cells to regenerate the defective bone by creeping substitution [3, 4]. Ceramics, polymers, metals, and composites have been successfully used for matrix-based bone engineering. Besides being biocompatible and osteoconductive, the microstructure and mechanical properties of the used scaffold should resemble normal bone. An ideal scaffold should be able to repair bone defect and to restore bone tissue function to the greatest extent [5–7].

Hydroxyapatite (HA) [Ca_10_(PO_4_)_6_(OH)_2_] is a major inorganic component of bone that has been used extensively in bone regeneration [3]. It is bioactive, biodegradable, and osteoconductive however, its weakness and brittleness restricted its clinical application in orthopedic surgery. Enhancing the mechanical properties of HA gained much attention [3, 8]. The nano-sized HA prepared by mechano-chemical synthesis, combustion preparation, or wet chemistry techniques has been used to increase HA surface area simulating the inorganic phase of normal bone [9, 10]. Innovations have made effort to advance both the resorbability and mechanical properties of tricalcium phosphate along with osteoconductive properties for more rapid bone regeneration rate of the HA [11] which gave rise to the development of the biphasic calcium phosphate (BCP). Studies revealed its impending in bone healing through osteoinduction and osteoconduction courses [12, 13]. Doping of BCP with cations such as Ag^+^, Cu^2+^, Zr^4+^ and Zn^2+^ results in substitution the Ca^2+^ in the calcium phosphate structure which have great effect on crystallinity, morphology and lattice parameters [14].

Zirconia (ZrO_2_) is a bio-inert, non-resorbable metal oxide that has been used extensively in dental implants and prosthetic devices because of its good mechanical and chemical properties [3]. It has excellent resistance to corrosion and high wear resistance, high bending strength and fracture toughness. Zirconium (Zr) is a naturally occurring trace element in the bone tissue. It is proved to be biocompatible osteoconductive material that can enhance osteoblastic proliferation and differentiation via bone morphogenetic protein/Smad Signaling Pathway (BMP/Smad) [10, 15].

The aim of the present study was to evaluate osteogenic potential and biocompatibility of doping biphasic calcium phosphate with zirconia nanoparticles compared to pure biphasic calcium phosphate for reconstruction of induced mandibular defects in dog model. Evaluation criteria included cone-beam computed tomographic and histopathologic evaluation.

## Materials and methods

### In vitro study

#### Preparation of graft material

Biphasic calcium phosphate (BCP, TCP/HA) and 4Zr TCP/HA were prepared to be grafted within mandibular defects. BCP was prepared from phosphogypsum (PG) (an industrialized waste material frequently prepared of gypsum matrix (CaSO_4_.2H_2_O) with other impurities) according to our previous study [16]. Pure Ca_10_(PO_4_)_6_(OH)_2_ was doped with Zr [Ca_10–*x*(Zr)_ (PO_4_)_6_(OH)_2_] [(*x*_Zr_ = 0 and 0.4) by wet precipitation technique where PG and zirconyl chloride octahydrate (ZrOCl_2_.8H_2_O) were mixed under dynamic stirring in distilled water. The pH of the solution was adjusted using ammonium hydroxide (NH_4_OH) until it reached 11. The reaction was monitored by addition of phosphoric acid (H_3_PO_4_), the temperature was attuned at 80 °C. The resulting precipitate was washed several times using deionized water before being filtered. The obtained powder was dried for 5 h at 100 °C then calcined in an oven for 2 h at 900 °C [17]. Powder was sieved into fine form of <0.037 mm and shaped into discs of 15 mm diameter using uniaxial pressure (20KN) and (7%) PVA solution as a binder. Then discs were sintered at 900 °C for 2 h before being characterized using X-ray diffraction analysis (XRD), surface area, antibacterial activity and cytocompatibility analysis.

Symbols of prepared samples and their implication is demonstrated in Table 1.

**Table 1:** Symbols of samples and their Implication

Symbol	Implication
TCP/HA	Biphasic of tricalcium phosphate and hydroxyapatite
4Zr TCP/HA	TCP/HA doped with zirconia nanoparticles with *x* value = 0.4

#### Characterization of graft material

Identification of the crystalline phases of the BCP powder before and after doping with zirconia were documented by (XRD) (BRUKER, D8 ADVANCED Cu target, Germany), CuKα radiations (*λ* = 1.5406 Å) using Ni filter. XRD diffractometer operated at 40 kV, 40 mA and 2°/min scan speed. Joint Committee on Powder Diffraction Standard (JCPDS) was used to recognize the designed phases [18]. The crystallite size (*D*) was measured based upon the Debye–Scherer’s equation:

$${{{{D}}}} = \left( {{{{{k}}}}\lambda /\beta \,{{{\mathrm{cos}}}}\,\theta } \right)$$

where *k* is the Scherer’s constant (*K* = 0.94), *λ* is the X-ray wavelength (1.54178 Å) and *β* is full width at half maximum (FWHM) of the diffraction peak.

The surface area, total pore volume and average pore diameter of TCP/HA were estimated employing the Brunauer–Emmett–Teller (BET) before and after zirconia doping. In total, 100 mg of the calcined powder was degassed and the adsorption desorption of nitrogen were conducted at −196 °C using a Micromeritics ASAP 2020 analyser (USA).

The antibacterial characteristics were tested using shake flask method to estimate the reduction percentage of the different microorganisms [three gram-positive bacteria [*Bacillus subtilis* (ATCC 6633), *Bacillus cereus* (ATCC 6629) and *Staphylococcus aureus* (ATCC 6538)], two gram-negative bacteria [*Escherichia coli* (ATCC25922) and *Pseudomonas aeruginosa* (ATCC27853] and an opportunistic pathogenic yeast [*Candida albicans* (ATCC 10231)], in comparison with TCP/HA and 4Zr TCP/HA after getting in contact with the tested samples compared to the number of bacterial cells surviving after 1-day incubation period [19]. Reduction % were expressed conferring to the following equation:

$${{{\mathrm{Reduction}}}}\left( \% \right) = \left( {{{{{A}}}} - {{{{B}}}}/{{{{A}}}}} \right) \times 100$$

where *A* is the number of microorganisms present in control flask containing pure bacterial strain and *B* represents the number of microorganisms in shake flask after applying the samples. Bacteria and yeast strains used are American Type Culture Collection obtained from the culture collection of the Department of Chemistry of Natural and Microbial Products, National Research Centre (NRC), Cairo, Egypt. The cell death mode of samples (25 mg disc/well) was deceived at two exposing time (24 and 72 h) to the MG-63 cells (1 × 10^4^ cells), scattered on cell culture slides. At the end of the incubation periods, all slides were washed with PBS, stained by ethidium bromide/acridine orange for 10 min then microscopically inspected via fluorescence microscope. The cytocompatibility information were published elsewhere [17].

### In vivo study

#### Animals

Twelve skeletally mature male mongrel dogs aging 18.9 ± 3.2 months and weighing 22.3 ± 2.1 kg were included in the study. All dogs underwent thorough clinical and hematological examinations to rule out systemic or bone disease. Dogs were quarantined for 2 weeks for acclimatization and to receive regular anti-parasitic medication. Animals were kept separately in individual cages with free access to water and were fed twice daily. All study procedures were approved by Animal Care and Use Ethical Committee of the National Research Centre (approval number#19096).

#### Study design

Randomized controlled experimental study was designed where three surgically induced cylindrical critical-sized (15 mm diameter × 4 mm depth) mandibular defects were created into the right and left side of the mandible. Mandibular defects were randomly allocated into one of the following groups:

- Control group: where the induced defect was created and left empty.
- TCP/HA group: where the induced defect was grafted with tricalcium phosphate/hydroxyapatite disc.
- 4Zr TCP/HA group: where the induced defect was grafted with tricalcium phosphate/hydroxyapatite doped with zirconia nanoparticles disc.

Dogs were evaluated clinically at 2-week interval till the end of the study at 12 weeks. Dogs were euthanized for radiographic and histopathologic evaluation.

#### Anesthetic protocol

The skin over the right and left mandibles was prepared for aseptic surgery and the cephalic vein was cannulated. Atropine sulfate 0.1% (Atropine Sulfate®, El Nasr pharm. Chem. Co. Egypt) was administered subcutaneously 15 min prior to anesthesia at a dose of 0.05 mg/kg body weight, and xylazine HCL 2% (Xylaject®, ADWIA Co. Egypt) was administered intramuscularly at a dose of 1 mg/kg body weight to calm the dogs. Ketamine HCL 5% (Ketamine®, Rotex Medica, Germany) was injected at a dose of 10 mg/kg body weight to induce anesthesia. Thiopental sodium 2.5% (Anapental®: Sigma-Tec, Egypt) at a dose of 25 mg/kg body weight was used to maintain anesthesia.

#### Induction of critical-sized defect

**Fig. 1:**
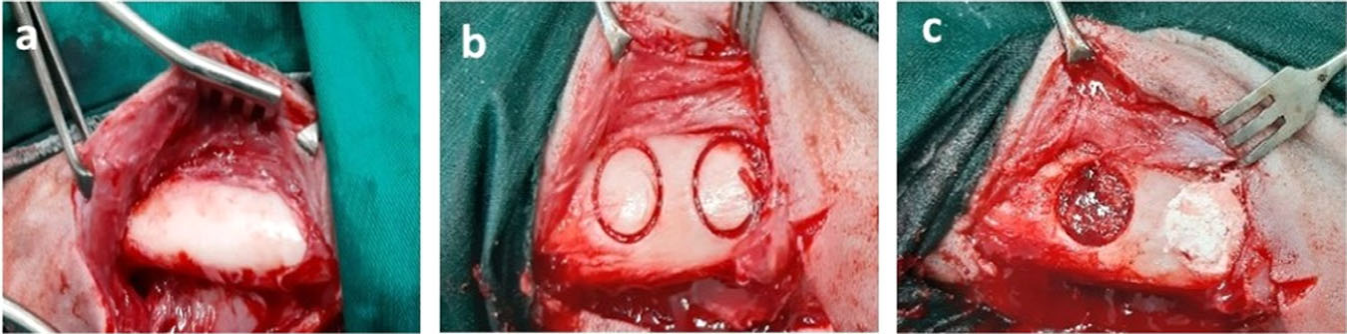
Intra-operative photograph demonstrating complete exposure of the mandible with elevation of the periosteum (**a**), induction of 15 mm circular bone defect (**b**) and grafting of the defect with tricalcium phosphate/hydroxyapatite disc with or without zirconia while control defects were kept empty without grafting (**c**)

An extra-oral approach was used to induce a circular critical-sized mandibular defects. Approximately 1 cm below the inferior border of the mandible, a 6 cm skin incision was made, and the deeper fascia was dissected to reveal the mandibular body. The periosteum was horizontally incised and elevated using a periosteal elevator. A 15 mm circular defect was made using a trephine bur (Trephine drill, Friatec AG, Germany) (Fig. 1). Irrigation was kept up to prevent bone heat necrosis during drilling. The inferior alveolar canal was carefully avoided during drilling. The prepared discs were inserted into the defects based on animal grouping (Fig. 1).

#### Post-operative care

Daily dressing of the surgical wound was done for 10 days using povidone iodine 2% solution. Systemic course of antibiotic (Ceftriaxone® 1000 mg i.m., Novartis Co., Sandoz, Switzerland) was prescribed at a dose of 1 gm /dog for 7 days following surgery. Skin sutures were removed 12 days following surgery.

Dogs were examined daily to evaluate surgical site (edema, hyperemia and swelling) and general health condition (appetite, body weight, clinical behavior, physical oral functions, mastication and deglutition).

#### Euthanasia and sampling

At the end of the study (12 weeks), sodium pentobarbitone 200 mg (Eutha-naze®, the premier pharmaceutical Co., office park Sloane, Bryanston 2125) at a dose of 2 ml/kg was used to humanely euthanize dogs. Right and left mandibles were dissected and disarticulated immediately after euthanasia for cone-beam computed tomography (CBCT) examination.

Bone samples including the induced mandibular defect as well as the surrounding tissue was harvested, labeled and sent for blind histopathologic evaluation. Liver and kidney tissue samples were harvested from all dogs.

#### Cone-beam computed tomography (CBCT) analysis

I-CAT machine was used with the following specifications (size of reconstructed volume diameter 16 cm-custom, resolution 2 voxel 26.9 s, exposure factors 37.07 mAs, 120 KVP, acquisition time 26.9 s). 3-D and panoramic views were captured as general views outlining and localizing the control and grafted mandibular defects. Cross-sectional views were taken as sections (in 2 rows) from buccal to lingual sides for description of the 1.5 mm thick slices through the defects to analyze bone healing, behavior, integration with the disc, inferior alveolar canal, and biomineralization in ten sequential sections (5 sections/row) passing posterior to anterior of the defect in the mandible.

Incorporated software program of I-CAT machine was used for quantitative mean bone density measurements of all CBCT scans. A constant area was selected for sagittal and coronal views (193 and 53.5 mm^2^ respectively) and ten readings of radiodensity (Hounsfield; HU statistics) were collected for each view with slice thickness 0.4 mm. The mean bone density values were calculated and expressed as area density in Hounsfield (HU) units.

#### Histopathologic and histomorphometric examination

Bone samples were sectioned in halves along the midline a bucco-lingual direction, fixed in 10% buffered formalin, decalcified using 15% formic acid, routinely processed, sectioned longitudinally in a bucco-lingual direction at 5 µm thickness. Liver and kidneys samples were fixed in 10% buffered formalin, routinely processed, and sectioned at 4 μm. Samples were stained with Hematoxylin and Eosin (H&E) and examined using the light microscope (Olympus CX 41, Germany).

Histomorphometric analysis was performed on digital images obtained using digital image analysis software (Olympus Soft Imaging Solutions GMBH, Model # LC20, Germany), analyzed using ImageJ (ImageJ ^®^ 1.53e, USA) image analysis software. The area percentages (%) of bone maturation within each defect were recorded.

#### Statistical analysis

Data were tabulated and presented as mean ± SE and range. Normality of distribution of the obtained measurements was tested using Kolmogorov–Smirnov test. A one-way analysis of variance (ANOVA) test was used to compare measurements of all groups. When statistically significant differences were detected, a Tukey post hoc test was used to determine significant differences between groups. Differences were considered statistically significant when *p* ≤ 0.05. All analyses were made using Statistical Package for Social Science, SPSS software, 20 (IBM SPSS Statistics^®^, Chicago, IL).

## Results

### Material characterization

X-ray diffraction analysis of TCP/HA indicated the main peaks conforming crystalline phase of β-TCP (JCPDS PDF no. 09-169) supplemented with that of HA (JCPDS PDF no. 09-432). XRD of 4Zr TCP/HA revealed new peak related to zirconia (ZrO_2_) (JCPDS PDF no. 01-81-1544) that was detected at 2*θ* = 30.18°. The crystallite size in nm and the ratio of TCP/HA of the prepared samples is demonstrated in Table 2.

**Table 2:** Crystallite size and TCP/HA proportions

Sample	Crystallite size(nm)	TCP/HA
TCP/HA	43.30	38.3:56.9
4ZrTCP/HA	56.99	42.3:47.6

The surface area and total pore volume was decreased by doping with zirconia as elucidated in Table 3.

**Table 3:** Surface area, total pore volume and average pore diameter of tested samples

Sample	BET surface area (m^2^/g)	Total pore volume (cm^3^/g)	Average pore diameter (nm)
TCP/HA	16.85	2.043 × 10^−2^	1.93
4Zr TCP/HA	11.87	1.489 × 10^−2^	1.93

Antibacterial activity revealed that Gram-positive and negative bacteria have unfavorable charged cell dividers which have an opportunity to impact the cell wall of the microscopic organisms.

Cytological compatibility of prepared materials revealed that the investigation of cell viabilities was signified in the form of mode of cell death next to the experience to the MG-63 bone-like cells (1 × 10^4^ cells). It was recognized that most of the cells (up to 95%) is remaining after 1 day, which is very close to normal cell viability. While upon increasing the incubation time to 72 h, they slightly decreased the percentages of intact cell even for control (normal cells alone). Reduction % of bacterial strain cells after incubation for 1 day is represented in Table 4.

**Table 4:** Reduction (%) of bacterial strain cells after 1-day incubation

Test bacteria	TCP/HA	4Zr TCP/HA
*Bacillus subtilis*	34.62%	70.76%
*Bacillus cereus*	65.81%	44.52%
*Staphylococcus aureus*	65.42%	55.33%
*Escherichia coli*	53.27%	23.36%
*Pseudomonas aeruginosa*	60.12%	64.72%
*Candida albicans*	62.47%	71.74%

### Clinical examination

All skin sutures were healed within 12 days following surgery. No local or systemic post-operative complications or adverse inflammatory/foreign-body reaction was recorded in any dog for 12 weeks follow-up.

### CBCT examination

#### Control group

At 12 weeks, a circular radiolucent gap with ill-defined hazy margin were seen within control defects. This gap was fused with the inferior alveolar canal (IAC) and violated the canal at the depth of the defect. No evidence of buccal cortical rim formation around the IAC at the depth of the defect. Incomplete corticalization of the area close to the distal root of the adjacent teeth was recorded. Faint radiopaque peripheral bone formation was seen along with the whole defect margin with subperiosteal peripheral buccal bone formation indicating thin, slender new buccal plate of bone. The mean defect size was 11.7 ± 0.4 × 3.5 ± 0.1 mm in diameter.

#### TCP/HA group

Circular radiopaque discs were seen within mandibular defects. There was reduction in size of the disc where it was resorbed from the periphery and inferior surface. A radiopaque margin of new bone was seen invading the disc making intimate contact and reduced interface between the disc and surrounding bone.

The disc was smoothly and perfectly matching the curvature of buccal plate of bone. Almost complete corticalization of the buccal cortical rim of IAC crossing the depth of the defect under the disc at the mesial or anterior half while there was incomplete corticalization in posterior or distal half. The mean defect size was 11.0 ± 0.3 × 2.2 ± 0.3 mm.

#### 4Zr TCP/HA group

Radiopacity and radiodensity of 4Zr TCP/HA grafted defects was increased compared to control and TCP/HA groups. There was excellent intimate disc-bone contact with only minute gap at the interface. Thick bridge of bone was formed under the surface of the disc. Complete buccal corticalization of IAC was seen crossing the depth of the defect peripherally with incomplete corticalization of IAC at the middle of the defect. The disc was bounded with new bone along the entire defect margin that was smoothly matching the curvature of the buccal plate of bone. Subperiosteal bone formation above the disc was seen on the buccal surface of the defect site. The defect size was reduced in diameter 10.1 ± 0.1 × 2.4 ± 0.4 mm.

**Fig. 2:**
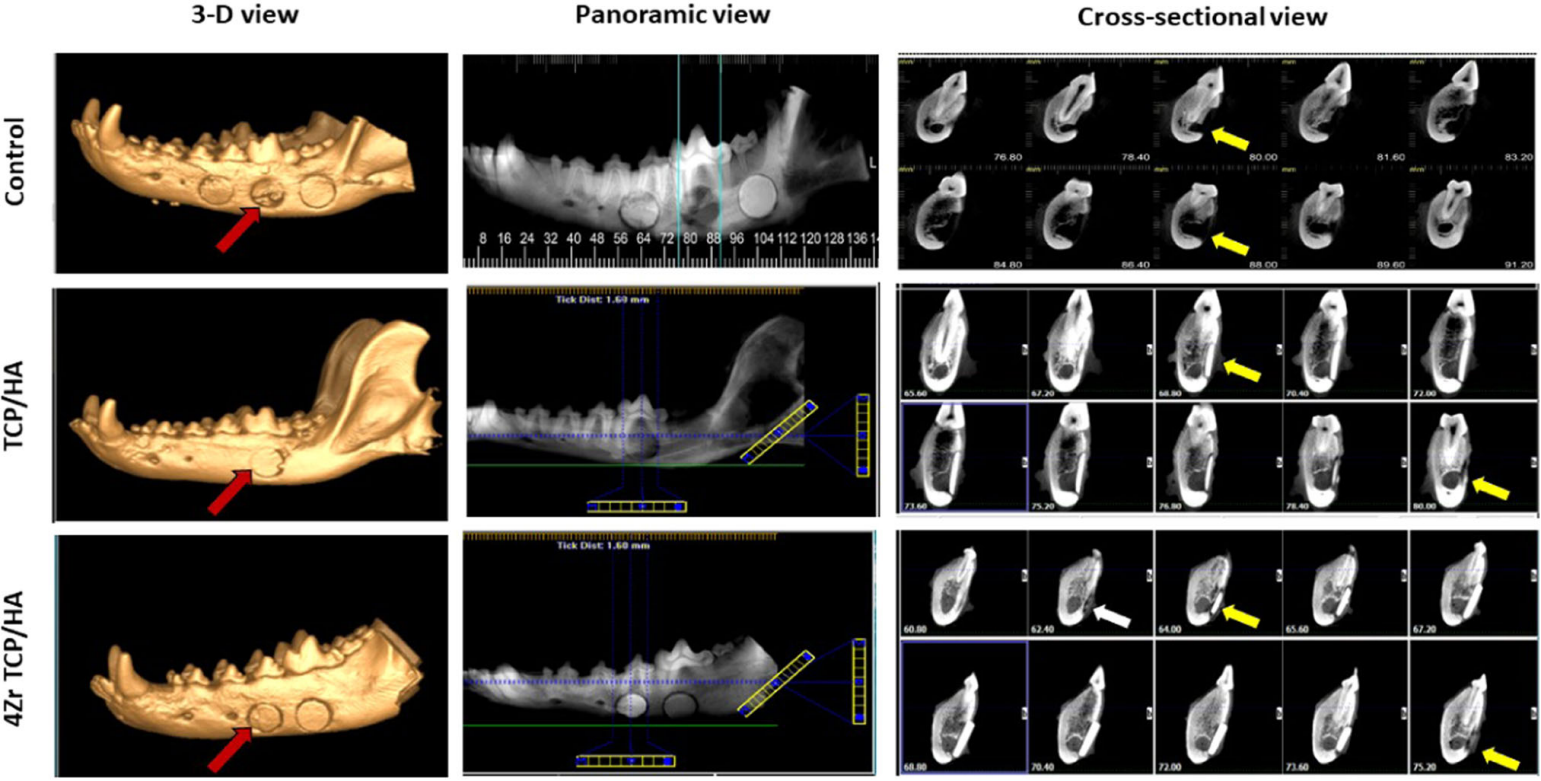
Three dimensional, panoramic and cross-sectional view cone-beam computed tomography of the induced mandibular defects at 12 weeks examination. Control defects (red arrow) demonstrated radiolucent circular defect with limited peripheral radiopacity without buccal rim corticalization (yellow arrow) around inferior alveolar canal (IAC). Defects grafted with tricalcium phosphate hydroxyapatite scaffolds (TCP/HA) (red arrow) demonstrated reduction in the size of the defect that was bounded by new bone formation with corticalization of the buccal rim (yellow arrow) around IAC. Defects grafted with zirconia-doped tricalcium phosphate hydroxyapatite scaffolds (4Zr TCP/HA) (red arrow) demonstrated reduction intimate contact with new bone formation with buccal rim corticalization around IAC (yellow arrow) with subperiosteal new bone formation (white arrow)

In all groups, no radiographic signs of bone infection or necrosis were seen in any of the defect as indicated by absence of periapical radiolucency around defect margins. All defects did not violate the vitality of the adjacent teeth. No evidence of migration or fragmentation of the grafted material into the submandibular region. Three dimensional, panoramic and cross-sectional view of CBCT scan of the studied groups is demonstrated in Fig. 2.

Quantitative measurement of bone area density as measured in Hounsfield (HU) units revealed statistically significant increase (*p* < 0.001) in bone density in TCP/HA and 4Zr TCP/HA groups compared to control group both in sagittal and coronal views.

Comparing TCP/HA and 4Zr TCP/HA groups, the increase in bone area density was statistically significant in coronal view (*p* = 0.002) and sagittal view (*p* = 0.05). Mean bone density as measured in sagittal and coronal CBCT scan of the three groups is demonstrated in Table 5.

**Table 5:** Mean bone density (and SE and range) as measured in sagittal and coronal CBCT scan of control, tricalcium phosphate/hydroxyapatite (TCP/HA) and zirconia-doped tricalcium phosphate/hydroxyapatite (4Zr TCP/HA) groups

View	Group	Mean	SE	Min	Max	95% confidence interval for Mean		*p* value	
						Lower bound	Upper bound		
Sagittal view	Control	328.3^a^	39.5	134.7	490.2	235.0	421.6	TCP/HA	0.000
								4Zr TCP/HA	0.000
	TCP/HA	1187.8^b,c^	44.9	1018.4	1328.9	1077.8	1297.8	Control	0.000
								4Zr TCP/HA	0.050
	4Zr TCP/HA	1600.9^c^	128.2	1087.2	1982.3	1271.4	1930.2	Control	0.000
								TCP/HA	0.050
Coronal view	Control	303.5^a^	33.9	194.7	475.6	223.2	383.7	TCP/HA	0.001
								4Zr TCP/HA	0.000
	TCP/HA	936.1^b^	73.0	679.3	1195.9	757.4	1114.7	Control	0.001
								4Zr TCP/HA	0.002
	4Zr TCP/HA	1575.9^c^	61.5	1394.5	1816.9	1417.8	1734.1	Control	0.000
								TCP/HA	0.002

Within each view, identical superscript letters indicate no statistically significant differences; different superscript letters indicate a statistically significant difference (*p* < 0.05)

### Histopathologic and histomorphometric evaluation

Control defects examined at 12 weeks revealed filling of the defect site with fibrous connective tissue with minimal osteoid formation.

The TCP**/**HA defects demonstrated the presence of dense connective tissue fibers in the defect areas intervening the graft material. Newly formed osteoid bone trabeculae within a fatty marrow and numerous blood vessels were seen along defects. Osteoblasts were seen within the margin and osteocytes were seen in their lacunae.

**Fig. 3:**
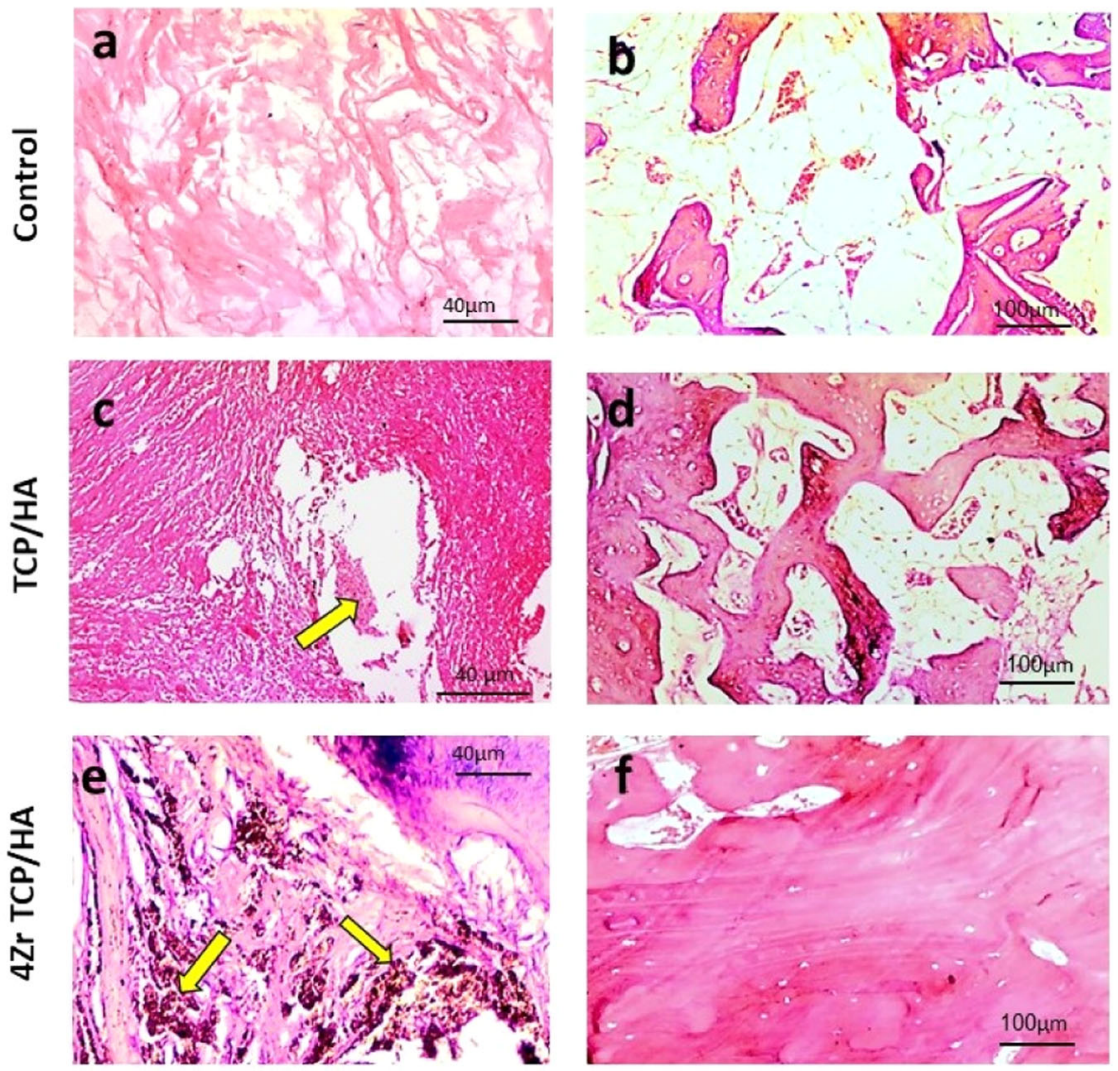
Photomicrograph of the mandibular defects in the studied groups stained with H&E. In the control group, the defect areas showed loose connective tissue (**a**) and osteoid tissue formation (**b**). In the TCP/HA group, the defects showed remnants of the graft material (yellow arrow) within a more condensed fibrous connective tissue (**c**), sections also showed newly formed haphazardly arranged bone trabeculae within a fatty marrow and blood vessels (**d**). In 4Zr TCP/HA group, remnants of the graft material (yellow arrows) were seen surrounded by dense fibrous connective tissue (**e**), also more organized bone tissue was evident with bone marrow, blood vessels and osteoblastic proliferation (**f**)

The 4Zr TCP**/**HA defects revealed entrapment of the graft material within dense connective tissue fibers. More organized bone tissue was evident with fatty bone marrow, blood vessels and osteoblastic proliferation (Fig. 3).

**Fig. 4:**
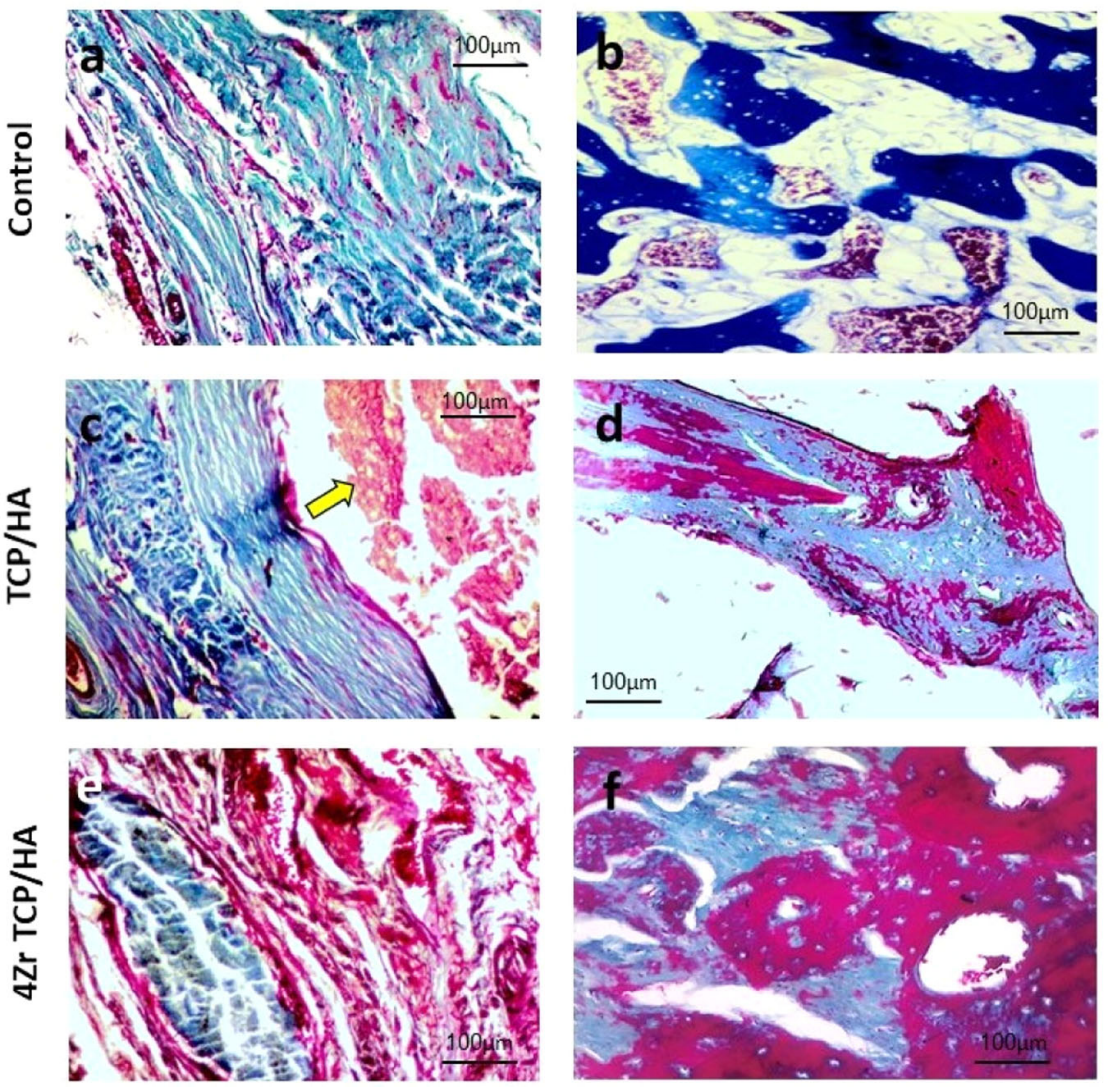
Photomicrograph of the mandibular defects in the studied groups stained with Masson trichrome. The control group showed loose connective tissue and immature bone (**a**) and formation of few spicules of osteoid bone tissue (**b**). The TCP/HA group revealed presence of remnants of the graft material (yellow arrow) in the defect site surrounded by connective tissue and newly formed bone (**c**), newly formed bony trabeculae showing slight maturation, with osteoblasts on their periphery and osteocytes in their lacunae (**d**). The 4Zr TCP/HA group showed the graft material enclosed within thin layer of fibrous connective tissue and blood vessels engorged with red blood cells (**e**), and showing noticeable bone maturation with more organized bone tissue, osteocytes in their lacunae and Haversian canals (**f**)

Masson trichrome staining revealed that control defects were occupied by loose connective tissue with only few trabeculae at the defect site. New bone formation was evident from the periphery to the center of the defects in 4Zr TCP/HA and TCP**/**HA groups that was stained with purple with Masson trichrome indicating complete maturation. Smaller trabecular spaces, and larger trabecular thickness were more evident in 4Zr TCP/HA compared to TCP**/**HA group. Spaces between fragmented graft material was occupied by the newly formed bone. In 4Zr TCP/HA, the newly formed bone was mature with presence of osteons and Haversian canals containing blood vessels and red blood cells. Osteocytes were seen inside their lacunae and osteoblasts were evident at the periphery of bone trabeculae (Fig. 4).

Statistically significant differences were recorded in bone area percent measured in histological sections of control, TCP/HA and 4Zr TCP/HA groups (*p* < 0.001) as demonstrated in Table 6.

**Table 6:** Mean and SD and range of bone area percent as measured from histological sections of control, tricalcium phosphate/hydroxyapatite (TCP/HA) and zirconia-doped tricalcium phosphate/hydroxyapatite (4Zr TCP/HA) groups

Group	Mean	SD	Min	Max	Difference	Mean difference	95% confidence interval for Mean		*p* value
							Lower bound	Upper bound	
Control	2.58^a^	1.78	0.41	5.41	TCP/HA	−14.1	−25.70	−2.31	0.001
					4Zr TCP/HA	−35.4			0.000
TCP/HA	16.58^b^	3.14	11.26	22.16	Control	−14.1	−47.08	−23.69	0.001
					4Zr TCP/HA	−21.4			0.000
4Zr TCP/HA	37.97^c^	11.83	22.03	61.02	Control	−35.4	−33.08	−9.69	0.000
					TCP/HA	−21.4			0.000

Different superscript letters indicate a statistically significant difference (*p* < 0.05)

Liver and kidney sections obtained from the three study groups revealed normal hepatic and renal tissue with no remarkable changes recorded in any of the examined sections.

## Discussion

The present study demonstrated that doping of zirconia into the biphasic calcium phosphate matrix promoted bone regeneration compared to un-doped biphasic calcium phosphate as indicated by CBCT radiographic and histopathologic evaluation.

X-ray diffraction analysis demonstrated that the development of zirconia phase had an impact on the proportion of the TCP/HA owing to the exchange of the larger radius ion of the Ca^2+^ (0.099 nm) by the smaller one of the Zr^4+^ (0.084 nm) in agreement with previous studies [17, 20] concluded that by increasing the TCP/HA proportion, their reactivity is intensified. El-Bassyouni et al. specified that such doping process was capable to improve the physicochemical, morphological and size parameters of the prepared material [21].

The minor dimension of the Zr^4+^ was reflected on that of the Ca^2+^. Doping with Zr^4+^ ions intensely impact the TCP/HA crystal size as the surface area of the TCP/HA is larger than that of the 4Zr TCP/HA.

Likewise, antibacterial activity obtained by combining zirconia and TCP/HA revealed a wide range of bactericidal effect because of their capability to produce reactive oxygen species, which ensure mechanical destruction of the bacterial cell. Previous study indicated that the formation of hydrogen peroxide (H_2_O_2_) from the zirconium-doped surfaces is a positive way for the inhabitancy of the bacterial growth [22]. Cell mode death supported the significance of Zr-doping. The increased viability of tested samples increases the likelihood of the materials to be applied in restoration of the damaged bone sites [17].

The use of biomaterials has evolved from being only biocompatible and bioinert substances replacing natural tissues to being functional bioactive materials that could have the ability to stimulate the human body for regeneration and healing or have antimicrobial purposes [23]. The use of animal models is mandatory to validate the bio-functionality, biocompatibility, biodegradability, osteointegrative, osteoconductive as well as osteoinductive properties of bone substitutes before being applied in clinical practice [24, 25]. The dog represents an ideal pre-clinical model for studying bone regeneration due to the great similarity between dog’s weight, size, bone density, bone microstructure and turn-over and that of human bone [26–28].

CBCT examination demonstrated that grafting mandibular defects with TCP/HA resulted in decreased defect size and increased bone density within the defect compared to control ones. The Ca and P content of HA promoted osteogenesis by increasing osteoblastic activity and differentiation. Additionally, the nano-sized HA acted as a nano porous scaffold for osteoblasts to form interconnected osteoid matrix and later mineralization [29]. Previous studies revealed that HA enhances osteoconductivity and bioactivity while lacking osteoinductivity [23]. Osteoinductivity could be achieved by adding dopants materials to HA [30]. In the current study, doping of TCP/HA with zirconia resulted in statistically significant increase in bone radiodensity compared to TCP/HA which could be attributed to the role of zirconia in increasing the mechanical properties (compressive strength and stiffness) as previously reported [17].

Combining TCP/HA and zirconia resulted in a synergistic action for effective osteoconduction and mineralization of osteoid tissue intermingled in the TCP/HA matrix scaffold and osteointegration with native bone. Doping with zirconia resulted in Ca/P ratio of 1.59 indicating the increasing of β-TCP phase in the presence of Zr ions as previously explained [31]. Similar results were reported in doping with Ag ions where the Ca/P ratio was 1.18 which resulted in formation of amorphous calcium phosphates and substitution of Ca ions by Ag ions inside the biphasic structure [17]. Additionally, the increase Ca/P ratio and TCP phase in zirconia doping resulted a bactericidal effect producing a microbial free microenvironment for enhanced osteogenesis.

Bone defect healing involves the reconstruction of the defect in multidimensional procedures with an overlapping timeline [1]. Histopathologic sections evaluating the osseous response following TCP/HA grafting demonstrated incomplete filling of the defect with osteoid tissue. Similar reports were reported in evaluation of pure HA beads as a graft material for treating induced bone defects in rabbits’ tibiae after 10 weeks [32].

Doping of TCP/HA with zirconia (4Zr TCP/HA group), both bone formation (as indicated by bone area percentage) and maturation (as confirmed by Masson trichrome staining) were significantly increased compared to TCP/HA group. The newly formed bone was mature and organized with more trabecular thickness and less trabecular space in between which could be explained by the role of zirconia in increasing osteoblastic proliferation and differentiation as previously concluded [33]. It has been reported that zirconia owns a great potential of inducing bone formation in biological environments. Zirconia coating was found to promote the build-up of apatite in simulated body fluids, which could induce proliferation and adhesion of osteoblasts, encouraging bone regeneration [34]. Single and co-doped TCP/HA with zirconia demonstrated deposition of apatite layers on their surfaces implicating their potential of bone regeneration [17].

In the current study, the presence of newly regenerated mature bone at the graft-bone interface as seen in histopathological sections of 4Zr TCP/HA group confirming Zr osteointegration properties. The presence of grafted material (zirconia) surrounded by dense fibrous connective tissue reflecting the non-resorbable, bio-inert, biocompatible criteria of zirconia. Similar results were reported following 12 weeks of implantation of zirconia graft material [35].

Cytocompatibility of zirconia-doped TCP/HA was previously tested on various cell lines including lymphocytes, monocytes, fibroblasts, osteoblasts and macrophage and in vivo models [17, 35]. In the present study, absence of local and systemic signs of acute or chronic toxicity was also documented. Histopathological sections of liver and kidney tissue obtained from all dogs did not reveal any remarkable histopathological changes till the end of the study at 12 weeks.

## Conclusions

The physicochemical, morphological and size parameters of nanocomposite prepared by combining zirconia and TCP/HA were improved. Bactericidal properties of zirconia-doped TCP/HA were increased which increases the likelihoods of its suitability to be applied in restoration of damaged bone in microbial free microenvironment with enhanced osteogenesis. Combining zirconia and TCP/HA resulted in a synergistic action for effective osteoinduction, osteoconduction and osteointegration with native bone. Future studies should be directed toward testing the long-term effect of combining zirconia and TCP /HA as matrix-based tissue engineered substitute and testing its applicability in clinical practice to restore damaged bone.

## Data Availability

The datasets supporting the conclusions of this article are available from the corresponding author upon reasonable request.
